# “Something that actually works”: Cannabis use among young people in the context of street entrenchment

**DOI:** 10.1371/journal.pone.0236243

**Published:** 2020-07-28

**Authors:** Braedon Paul, Madison Thulien, Rod Knight, M. J. Milloy, Ben Howard, Scarlett Nelson, Danya Fast

**Affiliations:** 1 Department of Medicine, University of British Columbia, Vancouver, Canada; 2 British Columbia Centre on Substance Use, Vancouver, Canada; 3 At-Risk Youth Study Peer Research Associate Team, Vancouver, Canada; Queens University Belfast, IRELAND

## Abstract

**Background:**

Cannabis is one of the most widely used substances among vulnerable young people (<26 years of age) experiencing street entrenchment. Although previous research has documented the role cannabis can play in harm reduction, substance use and mental health treatment and pain management, this research has predominantly been quantitative and focused on adult drug-using populations. Little qualitative work has examined how young people who use drugs understand, experience, and engage with cannabis in the context of street entrenchment and drug use trajectories that include the use of other substances such as alcohol, opioids and crystal methamphetamine (meth).

**Methods:**

Semi-structured, in-depth qualitative interviews were conducted between 2017 and 2019 with 56 young people recruited from a cohort of street-involved youth in Vancouver, Canada. We also conducted 13 interviews with 12 youth-focused care providers across the same time period. Interview data were triangulated by drawing on the findings of a program of anthropological research conducted by the senior author since 2008. Interviews were transcribed verbatim and thematic analysis was conducted.

**Results:**

The vast majority of study participants engaged in daily, intensive cannabis use at the same time as they cycled on and off other substances that were perceived as much more harmful (primarily alcohol, fentanyl, heroin and meth). While most participants derived significant pleasure from the use of cannabis, no participants in our study described using cannabis for purely recreational purposes. A number of participants explicitly framed cannabis as a form of mental health and substance use treatment that was more effective and “healthier” than the long-term use of psychopharmaceuticals and medication-assisted substance use treatment (e.g., opioid agonist therapies). Cannabis use was also understood to ameliorate some of the harms of, or even facilitate transitions out of, periods of street-based homelessness. While the majority of our participants highlighted the positive effects of regular cannabis consumption, some described how intensive cannabis use could generate significant harms.

**Conclusion:**

In the context of the recent legalization of non-medical cannabis use in Canada and amid ongoing overdose and housing crises, it is imperative that future policy and programming interventions and provider education and training be responsive to the ways in which vulnerable youth in our setting are actively using cannabis to navigate their everyday lives and healthcare needs.

## Introduction

In Canada, it is estimated that there are between 35,000 and 45,000 young people who are living without a stable or safe residence [1]. In Vancouver, a recent homeless count revealed that there are nearly 700 youth aged 13 to 24 living on the streets of Greater Vancouver [2]. Youth experiencing street entrenchment (i.e., those experiencing homelessness or without stable housing, frequently in the context of other kinds of overlapping exclusion along axes of race, class, sexual orientation and gender identity) are vulnerable to a variety of health and social harms [3], including elevated rates of substance use [4], mental illness [5], blood-borne and sexually transmitted infections [6, 7], poor nutrition [1] and exposure to physical, sexual and emotional violence [1, 8]. Street entrenched youth are also vulnerable to fatal and non-fatal overdose, particularly in the context of the current crisis driven by the proliferation of illicitly-manufactured fentanyl, related analogues and fentanyl-adulterated stimulants [9]. In the province of British Columbia, illicit drug overdose deaths among young people between the ages of 10 and 18 increased from 3 deaths in 2014 to 18 in 2018, while overdose deaths among young people aged 19 to 29 increased from 83 to 300 across the same time period [10]. The provincial government declared overdose deaths a public health emergency in April 2016 [11].

Amid intersecting crises of homelessness and climbing overdose rates, Vancouver continues to be recognized as an international leader in the rapid implementation of numerous interventions to reduce drug-related harms. In the downtown core, services such as needle distribution programs, supervised injection facilities and overdose prevention sites, the widespread distribution of take-home naloxone kits and the availability of opioid agonist therapies (OAT; e.g., methadone, buprenorphine/naloxone) have been paramount in reducing drug-related harms [5, 12, 13]. Across Greater Vancouver, various public health stakeholders are currently expanding efforts to create a comprehensive substance use care system for vulnerable adolescents and young adults that spans acute and community healthcare settings, including clinics, hospitals, and residential detox, treatment and recovery settings. For example, the local health authority has recently rolled out a new Youth Intensive Case Management Team staffed by nurse practitioners and other healthcare professionals, which provides mental health and substance use care to youth across in- and out-patient community healthcare settings (e.g., drop-in centers, residential treatment centers) [14].

And yet, in this setting service providers continue to struggle to connect young people, and particularly youth experiencing street entrenchment, to care. A largely quantitative body of research from settings across North America has highlighted various barriers to accessing care among vulnerable youth, including long wait times, age restrictions, a lack of trained providers and access to pharmacotherapies, and experiences of discrimination on the basis of gender, race and sexuality [15–19]. For young people experiencing street entrenchment, barriers to services are often compounded for those experiencing the concurrent disorders of substance use and mental health issues [20], and residential instability and mobility across institutional (e.g., government care homes, correctional facilities) and non-institutional (e.g., ‘flop houses’) settings over time [21, 22]. Moreover, among vulnerable youth, negative experiences with various forms of institutionalization and care stretching back to their childhoods can lead them to the conclusion that reducing or eliminating drug use are things best accomplished independently, without professional help [23].

Previous work by our team in Greater Vancouver has demonstrated that one of the emic strategies street entrenched young people may employ to independently mediate their drug use is the use of one substance to transition away from another. For example, many youth in our setting begin using crystal methamphetamine (meth) in order to reduce or eliminate what they view as increasingly problematic crack cocaine and opioid use [23]. Preliminary work by our team similarly demonstrates that young people may use cannabis to reduce the harms caused by other forms of substance use [24] and in order to transition away from more harmful forms of substance use (e.g., injection drug use; see Boyd et al. [25]).

In Canada, cannabis is one of the most widely used substances, particularly among young people. Health Canada reports that cannabis use among youth ages 16 to 25 is over two times greater than among adults [26], and UNICEF reports that Canadian youth have some of the highest rates of cannabis use in the world [27]. Although previous research has documented the role cannabis can play in harm reduction, substance use and mental health treatment and pain management, this research has predominantly been quantitative and focused on adult drug-using populations [28–33]. Less research has examined qualitatively the therapeutic uses of cannabis among youth, particularly youth experiencing street entrenchment (see Bottorff et al. [34] for an exception). Research by Jenkins and colleagues [35, 36] underscores the need to consider these kinds of social spatial contexts in order to understand how and why young people use cannabis, and tailor the design and delivery of substance use and harm reduction interventions to meet the needs of specific youth populations. A more nuanced understanding of cannabis use among youth in the context of street entrenchment is particularly important given the markedly widespread use of cannabis use among this population. Although the non-medical use of cannabis by people older than age 18 has been legal and regulated in our study setting since October 2018, cannabis has long been highly accessible and relatively affordable among local youth due to thriving street-based drug scenes and illicit retail storefronts and other outlets that pre-date the regulatory changes [37]. Studies from British Columbia and Vancouver have estimated the prevalence of cannabis use among street-involved youth to be as high as 98% [38, 39], while nearly 20% of youth in this setting report having sold cannabis within the past six months [40]. We undertook the present study in order to examine how young people understood, experienced and engaged with cannabis in the context of drug scene entrenchment and drug use trajectories that included the use of other substances such as alcohol, fentanyl, heroin and meth. Such research is of particular importance in a setting where public health crises of homelessness and an ongoing overdose epidemic have the potential to significantly shape youth’s perceptions and practices surrounding cannabis, as well as the way that stakeholders respond to them.

## Methods

Seventy-eight semi-structured, in-depth qualitative interviews were conducted from March 2017 to August 2019 with 56 young people between the ages of 16 and 26 (31 youth completed one or more follow up interviews). Youth were recruited from the At-Risk Youth Study (ARYS), a prospective cohort of over 1,000 street entrenched young people who use drugs that has been described in detail elsewhere [41]. We also conducted 13 interviews with 12 youth-focused care providers (one provider completed a follow up interview), including six family physicians, one nurse practitioner, one nurse, two drug and alcohol counselors, and two social workers. Service providers were recruited by the last author (DF), drawing on her ongoing relationships with those working in the field of youth substance use treatment and care.

Interviews were undertaken by a medical anthropologist (DF) and a research coordinator (MT) trained in qualitative interviewing and facilitated with semi-structured interview guides. Interviews with youth were designed to elicit broad discussions of their substance use and care trajectories, including experiences with and perspectives on cannabis use. For interviews with providers, we sought to elicit their experiences with and perspectives on the rapidly transforming substance use care landscape in Vancouver. While we did not explicitly raise cannabis use among street entrenched youth during these provider interviews, this emerged as an important theme in several conversations. All study participants provided their written informed consent, and youth participants were compensated with a $30 honorarium for their time. We received ethical approval from the University of British Columbia/Providence Health Care Behavioral Research Ethics Board to recruit and interview young people 14 years of age and older as emancipated minors, and therefore no parental or guardian consent was required for any of our youth participants.

Interview findings and emerging analyses for this study were triangulated by drawing on the findings of a larger program of anthropological research conducted by the senior author since early 2008 with street entrenched youth and their providers in Greater Vancouver. This research program has included a focus on tracing young people’s substance use and care trajectories across time and place, and hundreds of hours of fieldwork in the settings of youth’s everyday lives, including the places where they sleep, eat, socialize, work, and access services and systems.

As is common in qualitative and anthropological approaches, data collection and analyses occurred concurrently as the study progressed. Interviews were transcribed verbatim, anonymized, and checked for accuracy. ATLAS.TI software was used to code and manage the data. An initial codebook was generated by DF that captured broad emergent themes and analytic categories (e.g. “experiences with cannabis”). Subsequent fieldwork and in-depth interviews were used by the study team to refine the codebook through the addition of new codes (e.g., “cannabis as harm reduction”). Over the study period, evolving interpretations of the data were discussed with young people in the field by DF, and more formally during subsequent in-depth interviews conducted by DF and MT. In addition, the research team discussed the content of interviews and fieldnotes throughout the data collection and analysis processes. Two Youth Peer Research Associates (BH and SN) assisted with member checking emerging findings and final analyses. We use narrative excerpts from specific interviews and fieldwork encounters to highlight themes we identified across interview accounts and fieldnotes. All names appearing below are pseudonyms.

## Results

Youth interview participants included 30 young men, 21 young women, and five non-binary individuals (gender was self-identified by youth). The median age of youth participants was 21. Thirty-one participants self-identified as White, eight self-identified as Indigenous, two self-identified as African Canadian, one self-identified as Middle Eastern, one self-identified as Asian, 11 self-identified as being of mixed ethnicity, and two did not want to identify their race or ethnicity. Consistent with quantitative research from our setting [39], the vast majority of youth study participants engaged in daily, intensive cannabis use at the same time as they cycled on and off other substances perceived to be more harmful (primarily alcohol, opioids and meth). All of the young people who participated in this study had tried cannabis at least once. Interestingly, in the context of substance use trajectories that included “harder” drugs such as fentanyl, heroin and meth, as well as binge alcohol use, many young people did not view cannabis use as a form of substance use per se. For example, during periods of time when they were smoking cannabis or consuming edible cannabis products exclusively (i.e., not engaging in any other forms of substance use), many referred to themselves as “clean off drugs,” “sober” or “drug free.”

### It’s the best medication there is

While most participants derived significant pleasure from the use of cannabis, we noted that no participants in our study described using cannabis for purely recreational purposes. Among participants, cannabis’ pleasurable euphoric effects were frequently cited as relieving longstanding mental and physical health issues–most notably, depression, anxiety, attention deficit hyperactivity disorder (ADHD) and chronic pain. Twenty one-year-old Blake was using meth daily at the time of his interview. As they described:

Weed is very medicinal–it’s the best medication there is. It cures my hyperactivity. I also have scoliosis, and my back pain stops, too, when I use it. (Indigenous non-binary person)

A number of youth believed strongly that regular cannabis use was preferable to the long term use of the various psychopharmaceuticals they were regularly prescribed for mental health issues such as depression and anxiety. Twenty-four-year-old Zack had recently stopped using fentanyl and meth at the time of his interview (see below). He described how cannabidiol (CBD) pills would eventually allow him to stop taking psychopharmaceuticals for anxiety and depression:

I’m taking Gabapentin and, uh, Trazodone. I’ve got anxiety and depression, but I don’t wanna be taking pills my whole life. So I’ve been buying the CBD pills a lot. It’s healthier. (White male)

Beyond the management of mental and physical health issues, a few young people also described using cannabis to ameliorate some of the harms of street-based homelessness. Nineteen-year-old Jeremiah had recently undergone treatment for cocaine use at the time of his interview. He had been struggling with multiple periods of homelessness and unstable housing during the past year. Jeremiah explained:

I’ve been sleeping on the beach for three months. I smoke a lot of weed. And then I don’t have the sadness. My environment is still sad, but smoking weed helps me a lot. That’s very, like, therapeutic for me. And it just lets me, like, keep going. (African Canadian male)

Other youth connected cannabis use to periods of relative stability in their lives, during which they were able to transition out of homelessness and problematic substance use. Twenty-one-year-old Carla was using meth daily at the time of her interview in 2018. Reflecting on the previous year, she described how smoking cannabis allowed her to move out of daily binge alcohol use and homelessness and into a period of relative stability in her life:

There was a period of time there when I was sober for about–almost 5 months. I still had cravings [for alcohol] but it wasn’t so bad because I was using pot to wean myself off of it. At least I had something, and then [on pot], you know, I’m not flailing off and not remembering anything and a week later I’m in a ditch somewhere–like, have none of my belongings, don’t know what city I’m in. Eventually I was able to maintain a job, I was able to move back with my dad. It’s much better, smoking pot, eating food, going to bed. Like, I just get too relaxed and lazy to want to do anything else. (White Female)

### It actually works

Although we did not explicitly question young people about the “gateway drug” hypothesis, they often questioned this argument, describing how cannabis use was not a route to “harder drugs” in their context. When engaging with this debate, youth were not so much questioning the order in which their substance use had initially progressed (e.g., from trying cannabis to experimenting with “harder” substances such as meth and opioids) as they were questioning the idea that cannabis use propelled them towards greater drug-related harms. Rather, in the context of broader substance use trajectories that included the intensive use of opioids, meth and alcohol, many young people described cannabis as a means of intermittently reducing their use of or eliminating these more problematic forms of substance use.

Twenty-one-year-old Gordy was injecting heroin and meth at the time of his interview and indicated that he had no intention of quitting either at that time. However, he did describe how smoking cannabis was an important form of “harm reduction” for when he was trying to use less heroin because it staved off opioid withdrawal:

It’s like harm reduction for me. When I’m sick and I have to have down [heroin], I could take a few bong rips instead and I’m, like, ‘Okay, I’m good.’ If I smoke weed all day, like, I might not even notice that, like, there’s no heroin in me. (While male)

Other participants framed cannabis more explicitly as a form of “treatment.” They described periods of time when they had used cannabis to carefully “taper” their use of more harmful substances over the course of several weeks or even months, describing, like Gordy, how cannabis reduced the severity of withdrawal symptoms, and also prevented relapse by satisfying cravings. Zack described using a combination of methadone and cannabis to eliminate daily injection fentanyl and meth use (see also Socías et al. [32]). It is notable that Zack (as well as a number of the other youth we interviewed) used cannabis not only to transition away from what they viewed as more harmful substances, but also from what they viewed as more harmful routes of administration (e.g., from injection drug use to smoking):

I don’t wanna [inject meth and heroin] anymore. As soon as I think about it–like, right now, I’m kind of getting a craving for it. But right after [this interview], I’m not gonna go out and pick any up, I’m gonna go to my dispensary and pick up a joint and I’ll be all fine. The methadone and the weed just, like, connects together, and then all the cravings are gone. (White male)

Twenty-year-old Justin had recently stopped using heroin and meth at the time of his interview. He reflected:

I’m pretty baked, like, 100 percent of the day every day. Weed blocks the thought of doing dope. Instead of being like, oh, why don’t I just do dope? I don’t think about anything really when I’m high on weed. I’m kind of still taking my Suboxone, but the Suboxone doesn’t really do anything ‘cause the weed just blocks, like, the thought of doing dope. (Indigenous male)

Unlike Zack, who described how cannabis and methadone “connected together” to eliminate cravings, Justin and a number of other youth described how cannabis was far more effective than OAT for the treatment of substance use disorders. Young people frequently described using cannabis to taper off of OAT, or indicated that they planned to pursue the strategy of using cannabis to get off OAT after they had undergone treatment. Several youth argued that cannabis should be an official part of the substance use treatment system. Carla explained:

Like, I always believed, like, when I was in treatment–they should dispense pot to people here, you know? Like, people can’t just suddenly not have anything. You still need something to just kind of fucking just take the edge off and calm your nerves. And, like, it–there’s no harm in pot. (White female)

Indeed, a number of youth indicated that they avoided going to treatment because it would mean giving up cannabis. Twenty-year-old Devon was using meth and heroin at the time of their interview. They reflected:

I haven’t considered going to residential treatment because I can’t use weed there, you know? Because, like, that’s a medicine for me. It’s not a drug. So, like, that’s been a huge barrier in my getting clean and sober. It’s a challenge for those of us who use [weed] medically. (Indigenous non-binary person)

Some healthcare providers and treatment and recovery settings in Vancouver have been working with vulnerable young people to determine how to incorporate cannabis into substance use and mental and physical health treatment plans. A small number of youth, including Zack, described staying at a recovery house where they were allowed to smoke cannabis, so long as they were discrete about it. Several of the providers we interviewed highlighted the importance of engaging in conversations with youth about their cannabis use and discussing both the potential risks and benefits of cannabis in the context of physical and mental health and substance use issues. Providers emphasized the particular significance of these conversations in the context of the current overdose crisis. For example, one clinical addictions counsellor described:

We’re imposing beliefs, values and judgements instead of listening to the youth when they say ‘Well, when I smoke pot I don’t feel suicidal.’ I actually have a client right now who has a family member who’s saying, ‘You can’t smoke pot.’ So [the young person] stopped smoking pot and became more suicidal and is now in the hospital. And then another service provider said to them, ‘Well, from a harm reduction perspective, you know, we want you alive so if you need to smoke a little pot to help you not experience suicidal ideation then, you know, let’s do that.’ It’s complex, right? Especially right now, when kids are dying [from overdoses]. So I do a little bit of education with families: ‘This is why we say smoke pot if you need to, because we want the person alive and if that means they need to smoke some pot, fine. For right now, we want them alive so that we can work on the other issues that they want to work on.’

### You can get addicted

While the majority of our participants emphasized the positive effects of cannabis consumption, a smaller number of participants did highlight negative aspects of cannabis use. Unlike those who argued against a sort of “gateway drug” effect in the context of substance use trajectories that included the use of alcohol, meth and opioids, a few youth indicated that using cannabis could trigger cravings for the more intense high that accompanies these “harder” forms of substance use. For example, 24-year-old Marcus stated: “If I pick up weed I will pick up crystal meth, benzos–I know what can happen” (White male).

A small number of youth described developing a problematic dependence on cannabis, leading to various health and social harms. Nineteen-year-old Ana was using heroin and meth at the time of her interview. She reflected:

A lot of people say, like, you can’t get addicted to smoking weed. Like, you can. For me, it became, like, daily, habitual. I had to have it to cope, and if I didn’t have it, I felt irritable. I felt like something wasn’t right. I always wanted to be high. It became this thing where, like, I would only smoke it out of a bong with tobacco [known as doing “poppers”]. And I would start getting really sick from it. It was a really bad addiction to poppers. And I remember this one time last summer, I took a popper and I seizured out. The hospital said it was a drug-induced seizure. (Middle Eastern and White female)

Twenty-four-year-old Mason was injecting morphine daily at the time of his interview. He described how a previous addiction to cannabis had also caused him significant harm:

I used to puke up stomach acid all the time when I smoked pot. I’d just smoke and smoke and starve, too, sometimes. Like instead of buying food I’d buy $30 worth of weed and just get through [my stomach problems] with weed. (White male)

Mason eventually turned to methadone and morphine to help get himself off cannabis:

As soon as I started doing morphine [the stomach pains from smoking too much cannabis and not eating] went away. I actually lied and said I did heroin and then I got on methadone just to get off the pot use. That’s how bad it got. I wanted something to help me with the pot use.

Mason and other youth reflected on how seeking treatment for cannabis use could be challenging in the Vancouver context, where many people do not view cannabis as a “real drug” or cannabis dependence as a “real thing”:

People treated me like total crap when I was [in residential treatment] for pot. I mean, like, total shit. It was like, oh, well, that’s not a drug. You don’t need to be in treatment. And when I went there for morphine and shit they were just kind of like, whoa [i.e., that’s a “real” addiction].

The providers we interviewed generally acknowledged both the potential benefits and harms of cannabis use among youth experiencing street entrenchment, emphasizing the importance of remaining unbiased during conversations with young people about their cannabis. As one counsellor reflected:

I think it’s important to have an open conversation with people about what pot means to them, and I think all service providers need to be having these conversations. We need to be looking at the pros and cons and asking young people, ‘How is it for you? You’re smoking pot, are you okay with that, or do you want to make some changes?’ And I have clients who will say, ‘Well, you know, everybody’s saying that pot’s great and everything but it’s really harmed me and I want to stop using.’ And if that’s what they tell me then I work with them to help them stop using or cut down, set some goals, and then talk about what happens once they stop using. How is that gonna affect you and how can we work with that? Because there are going to be consequences to not using anymore, especially if you’ve been using to self-medicate. But if they say, ‘Hey, pot is what keeps me sane’ it’s like, okay, then let’s work on other stuff.

## Discussion

While some young people in our setting identified negative effects of regular cannabis use, our findings also underscore the therapeutic value of cannabis among many youth in the context of street entrenchment and substance use trajectories that include the intensive use of alcohol, opioids and meth. Despite reasonable access to health and substance use services in a setting like downtown Vancouver, many vulnerable youth instead elect to use cannabis to mediate substance use and mental and physical health issues, as well as to ameliorate some of the harms of, or even transition out of, periods of street-based homelessness. A number of participants explicitly framed cannabis as an effective form of mental health and substance use treatment. Participants used cannabis to reduce or achieve abstinence from what they perceived as more dangerous forms of substance use, and to transition away from harmful routes of administration such as injection drug use. They also used cannabis to transition away from psychopharmaceuticals and medication-assisted substance use treatment (e.g., OAT) that were generally viewed as less effective and “healthy” in the long term. This is consistent with other work by our team [42] which has demonstrated the undesirability of long-term OAT among a large number of street entrenched young people in our setting.

Our findings are consistent with previous studies focused on adult populations which demonstrate that cannabis use can reduce the frequency of prescription [31, 43] and illicit [30] opioid use among people who inject drugs, and can be used to transition away from alcohol, tobacco, crack cocaine, and various other licit and illicit forms of substance use among both street entrenched and non-street entrenched individuals [28, 32, 44]. Motivations for using cannabis as a substitute for other substances have included the limited side effects of regular cannabis use, a lack of painful withdrawal symptoms, and its efficacy in managing nausea, seizures, sleep disorders, depression, anxiety and ADHD [45–47]. Further supporting the value of cannabis as a form of substitution therapy is a 2016 US study, which reported that the use of prescription drugs for which cannabis could act as an alternative (e.g., painkillers) fell significantly in states following the implementation of medical cannabis laws [48].

Our findings also shed additional light on a recent quantitative study by Reddon and colleagues, which found a negative association between frequent cannabis use and initiating injection drug use among street entrenched young people in Vancouver [49]. Taken together, these findings challenge some of the assumptions built into the “gateway drug hypothesis,” particularly as these assumptions apply to vulnerable youth populations whose substance use trajectories include “harder” drugs such as fentanyl, heroin and meth, as well as binge alcohol use. The gateway drug hypothesis posits that the early use of cannabis, tobacco and/or alcohol directly increases an individual’s risk of progressing to more serious forms of substance use in adulthood [50]. Critiques of this hypothesis have emphasized the importance of genetic, psychosocial and environmental factors over the causal effects of a “gateway” substance in shaping harmful drug use trajectories [51–53]. While our findings do not speak to the chronological progression of young people’s substance use trajectories (e.g., from initiating cannabis use to experimenting with “harder” substances such as opioids and meth), they do demonstrate what could be referred to as a “reverse gateway effect” among some street entrenched young people, whereby cannabis use was associated with the intermittent reduction, elimination or prevention of more harmful forms of drug use such as meth and opioid use. This finding is particularly significant in the context of the current overdose crisis, which has claimed the lives of more than 1000 young people in the province of British Columbia since 2016 [10]. Further research is needed to understand how the use of cannabis among youth experiencing street entrenchment might be protective in our and other similar settings in the context of the current crisis. At minimum, we must acknowledge cannabis use as an emic harm reduction and treatment strategy that is commonly employed among some highly vulnerable youth to facilitate periods of transition away from what are perceived as more dangerous forms and modes of drug use, resulting in significant–albeit usually intermittent–reductions in risk and harm from both an emic and public health perspective [36, 54].

The use of cannabis as a form of intermittent harm reduction and treatment among many of our participants may reflect ongoing barriers to professional care among street entrenched young people in our setting. Previous research, including our own, has highlighted the numerous challenges vulnerable youth face when attempting to access substance use care, including the reluctance of many providers to provide OAT to youth [55], the physical location of services in heavy drug using (“triggering”) neighbourhoods [56], the undesirable expectations of particular programs [24], and a longstanding sense of distrust in “the system” [23]. As our previous research [23] as well as that of others [34] has shown, these barriers lead many young people to the conclusion that addressing substance use-related harms, as well as ongoing mental and physical health issues, is something that is best accomplished independently. Research approaches that bring together qualitative methods and implementation science are urgently needed to better adapt and design substance use and mental health services that are accessible, acceptable, appropriate and effective for young people experiencing street entrenchment in our and other similar settings [57]. Interventions must be contextually relevant and responsive to the perspectives and lived experiences of street entrenched youth [35], and work to address the marginalization and exclusion that many of them have experienced across their lives and rebuild their trust in “the system.” Service design and provider education and training must incorporate consideration of the current evidence regarding both the potential risks and benefits of cannabis use among this population, including the role that cannabis can play in intermittently or more permanently reducing or eliminating more harmful forms of substance use and mediating mental and physical health issues.

The youth who participated in this study cycled in and out of forms of substance use (e.g., daily injection meth and opioid use) that generally pose more immediate risks than daily, intensive cannabis use. However, some of our youth participants did emphasize the negative effects of intensive cannabis use. More research is needed to develop a clear picture of both the positive and negative effects of cannabis use among this population. Conducting research on cannabis, particularly with respect to its therapeutic possibilities among youth, is challenging for a variety of reasons [36, 58]. Consequently, the current evidence around the therapeutic value of cannabis is partial and mixed, particularly as it pertains to mental health issues. For example, while relief from anxiety and depressive symptoms are among the most widely reported reasons for therapeutic cannabis use [59], quantitative studies have shown dose-, frequency- and duration-dependent increases in rates of depression and anxiety among individuals who use cannabis, particularly if initiated at a young age [60–62]. Ongoing research has also identified an association between cannabis use and structural and functional brain alterations, particularly among adolescents [63, 64]. The risk of schizophrenia has been associated with chronic cannabis use during adolescence [65]; however, this association might be at least partially explained by a predisposition to cannabis use among individuals with genetic susceptibility to schizophrenia [66, 67]. The most frequently noted adverse effects of cannabis–that is, transient impairments in cognitive and psychomotor function [68, 69]–have been shown to desist during periods of abstinence from cannabis [70]. In sum, while various negative associations have been observed in previous research, we continue to have a poor understanding of what drives these associations and their clinical significance. Moreover, while intensive daily cannabis use during adolescence and young adulthood might indeed cause adverse health effects, these harms must be weighed against the harms associated with the intensive use of substances such as alcohol, meth, heroin and fentanyl–particularly in the context of the current overdose crisis.

In conclusion, our findings highlight the potential benefits and harms of cannabis use among young people in the context of street entrenchment and substance use trajectories that include the intensive use of alcohol, opioids and meth. While a small number of youth spoke to the potential dangers of cannabis use, the majority emphasized cannabis as a tool that allowed them to transition away from what they viewed as more problematic forms of drug use for periods of time, and to relieve longstanding physical and mental health issues. Particularly in the context of the recent legalization of non-medical cannabis use in Canada and amid ongoing overdose and housing crises, it is imperative that future policy and programming interventions and provider education and training be responsive to the ways in which youth in our setting are actively using cannabis to navigate their everyday lives and healthcare needs.
